# Breast conserving surgery using oxidized regenerated cellulose as filling biomaterial: recommendations to improve clinical outcomes

**DOI:** 10.1186/s12905-021-01436-9

**Published:** 2021-08-04

**Authors:** Gianluca Franceschini

**Affiliations:** 1 Multidisciplinary Breast Unit, Department of Woman and Child Health and Public Health, Università Cattolica del Sacro Cuore, Largo Agostino Gemelli, 8, 00168 Rome, Italy; 2 Fondazione Policlinico Universitario Agostino Gemelli IRCCS, Università Cattolica del Sacro Cuore, Largo Agostino Gemelli, 8, 00168 Rome, Italy

**Keywords:** Breast cancer, Breast conserving surgery, Hemostatic materials, Oxidized regenerated cellulose, Postoperative complications, Aesthetic results

## Abstract

Oxidized regenerated cellulose is considered an optimal local hemostatic agent thanks to its favorable biocompatibility, absorption characteristics and ease-of-use. Recently, oxidized regenerated cellulose has also been used as a filler in breast conserving surgery with a reconstructive purpose in order to repair partial breast defects and improve aesthetic outcomes. However, some postoperative problems due to its use, such as allergic reactions, seroma, foreign-body reaction and misdiagnosis during the follow-up of breast cancer patients, have been shown. These possible surgical complications can undermine clinical outcomes and lead to delay the beginning of oncological treatments with a negative impact on patient quality of life and survival. An adequate surgical expertise and the compliance with some specific recommendations are crucial in order to minimize postoperative issues and optimise aesthetic outcomes.

## Intoduction

I have browsed with interest the article of Li and colleagues published in “BMC Women's Health” [1] and would like to provide some useful remarks to optimise the clinical use of oxidized regenerated cellulose (ORC) as a filler in breast conserving surgery (BCS).

ORC is a bioabsorbable, sterile material prepared by the controlled oxidation of regenerated cellulose; It is composed of cellulose that is a homopolysaccharide of glucopyranose units polymerized together by beta [1–4] glycosidic bonds, regenerated to create organized fibers [2].

ORC is an optimal hemostatic agent that may be left in the surgical breast site to control bleeding thanks to ease-of-use and favourable biocompatibility [2, 3]. Once the ORC has been saturated with blood, it forms a black or brownish lump with a gel-like consistency that allows the clot formation, so acting as an adjuvant in the process of local hemostasis [2–4]. In addition to its local hemostatic properties, ORC has bactericidal activity thanks to its ability to reduce pH levels below 4.0, blocking bacterial growth and survival [2].

Recently, ORC has also been used as a filler in BCS with a reconstructive purpose in order to repair partial breast defects and improve the aesthetic outcomes [4–6].

Li et al. [1] have reported that “oxidized regenerated cellulose and gelatin sponge are feasible filling materials for partial breast defects” and can be useful in BCS in order to optimise cosmetic results; the evaluation of aesthetic outcomes, 6 months after surgery using the Harvard breast cosmetic grading scale, has documented very positive results in 18 patients in the ORC group and 15 patients in the gelatin sponge group [1].

Our previous clinical experience has shown that the ORC (Tabotamp Fibrillar® 10 × 10 cm, Johnson & Johnson; Ethicon USA) is a safe aid to minimize the risk of postoperative haematoma and optimise the cosmetic results in patients undergoing breast conservative surgery [5].

However, in order to perform a conscious and proper use of ORC in BCS, it is crucial to know its potential benefits but also some possible issues that can cause a negative impact on clinical outcomes; ORC can determine, like other biomaterials, allergic skin reactions, seroma as consequence of excessive digestion and foreign-body reaction with risk of extrusion due to its sub-optimal and inadequate absorption [4–7]. When ORC has been used in BCS, a significant postoperative seroma, high rate of red syndrome with acute dermatitis and eczema and some cases of foreign body reaction, that required surgical removal, have been reported [4–7].

These possible surgical complications can undermine clinical outcomes and lead to delay the beginning of oncological treatments with a negative impact on patient quality of life, survival and hospital costs.

Furthermore, specific radiological findings due to the fibrogenetic ORC-induced reaction and its partial absorption, can cause diagnostic mistakes and false alarms during the follow-up of breast cancer patients, if not properly interpreted; a previous experimental study on Wistar rats has analyzed the tissue reaction to locally implanted ORC and reported that its absorption is not always complete, resulting in biomaterial retention [4]; this trial has shown that tissues present chronic inflammation, central liponecrosis, neoangiogenesis (both centrally and periphery; homogenous vascularization) and diffuse fibrosis that is stable at postoperative week 30 when the ORC is used [4]; an excessive and improper fibrogenesis due to ORC can culminate in the creation of a three-dimensional fibrotic structure with a peculiar imaging and enhance the risk of diagnostic mistake during follow-up. The use of ORC may cause a granulomatous reaction that may mimic recurrent or progressive tumor, abscess, hematoma sequaele or area of fat necrosis on postoperative imaging studies creating a difficult challenge in differential diagnosis [2–4]; in our clinical experience with ORC in breast conserving surgery, postoperative mammography and ultrasound imaging have shown a typical lesion with circumscribed margins and internal hyperechoic nodules that we have called “ile-flottante” [7, 8]; breast magnetic resonance imaging (MRI) has confirmed a specific pattern with a well-encapsulated, hyperintense collection with circumscribed margins and internal hypointense nodules on the T2-weighted and short tau inversion recovery (STIR) sequences [9].

**Fig. 1:**
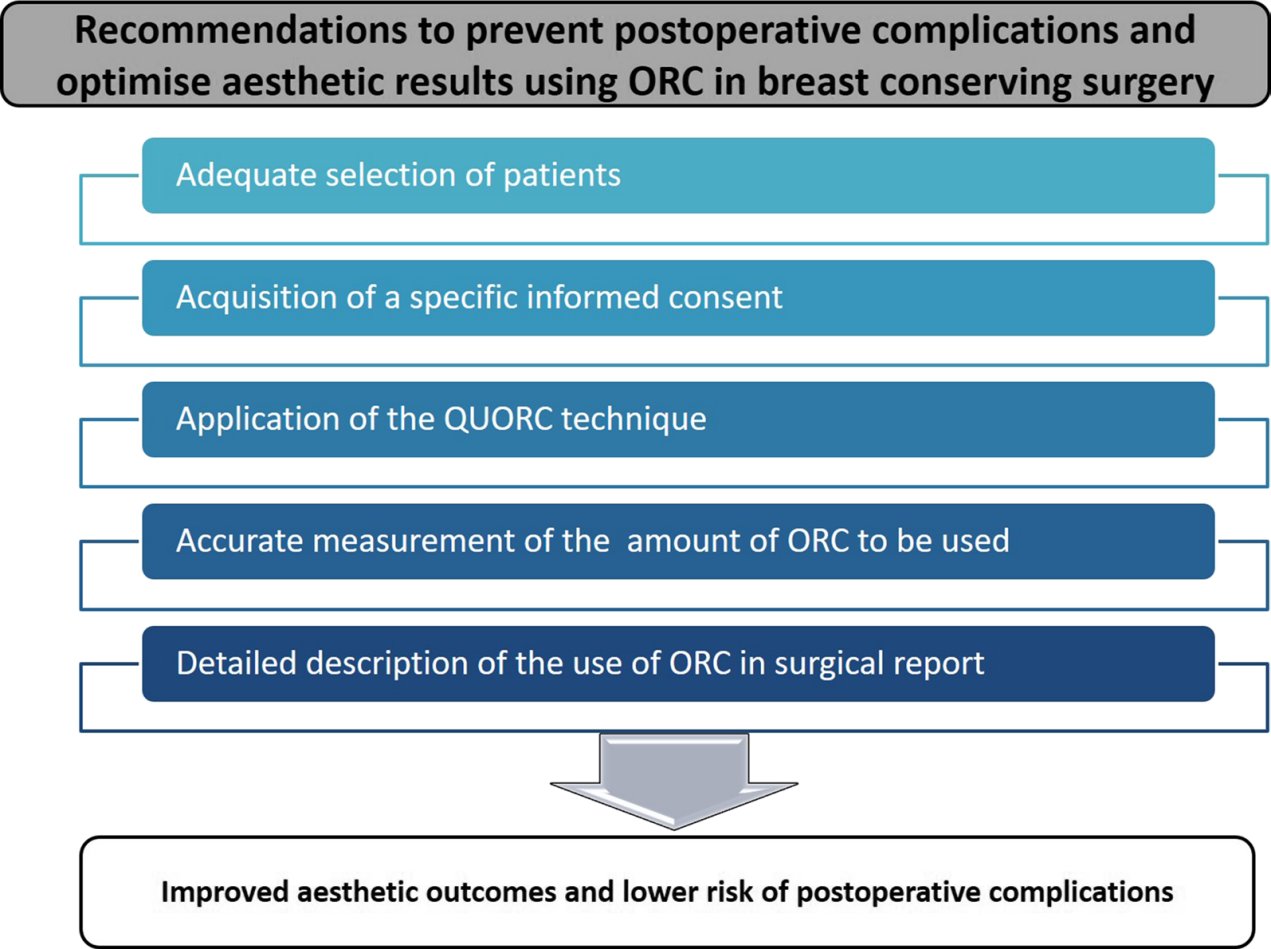
Principal recommendations to prevent postoperative complications and optimise aesthetic outcomes using ORC in breast conserving surgery (*ORC* oxidized regenerated cellulose, *QUORC* QUadrantectomy with oxidized regenerated cellulose)

In view of previous considerations, the breast surgeons should always follow some specific and standardized recommendations to optimise aesthetic outcomes and minimize postoperative issues (Fig. 1):

- Careful clinical assessment to select the adequate candidates to conservative surgery with ORC; this biomaterial should not be used as a filler in patients with specific comorbidities and higher risk of postoperative infections (e.g., immune diseases, non-controlled diabetes mellitus or after neoadjuvant chemotherapy).
- An innovative surgical technique called “QUORC” (QUadrantectomy with Oxidized Regenerated Cellulose) should be considered when surgical treatment is performed with use of ORC [10]: cutaneous incision should be performed away from cancer site in order to avoid the biomaterial being directly below surgical suture and reduce the risk of its extrusion; the maintenance of an adequate subcutaneous thickness should be ensured by an accurate dissection of gland in subdermal fascial plane in order to provide a safe coverage; accurate weight control of removed tissue should be realized to determine proper reconstructive volumes and carefully calibrate amount of ORC to be used as filler to prevent overdose; ORC pieces should properly fill the surgical cavity but should not be overblown to avoid excessive fibrosis and foreign-body reaction; a proper dissection of the residual gland from subcutaneous plane should create two parenchymal flaps that can be sutured together to cover ORC-filled cavity and secure the biomaterial.
- Antibiotic therapy should be prophylactically taken for at last 5 days in the postoperative period to prevent infections when surgical treatment is performed with use of ORC [5].
- Management of some postoperative complications should start as soon as possible with steroids and antihistamine medications in case of red breast syndrome; repeated percutaneous aspirations should be performed in case of seroma to quickly resolve the problem and to avoid delay in adjuvant therapies.
- The use of ORC should clearly be described in the surgical report so that breast radiologists can properly interpret the peculiar imaging due to this biomaterial and avoid diagnostic mistakes during the follow-up.

In conclusion, I agree with Li and colleagues that the application of ORC can be a feasible option in order to optimise aesthetic outcomes in breast conserving surgery as long as some specific and standardized tasks are always performed by skilled breast surgeons.

## Data Availability

Not applicable.

## Abbreviations

ORC: Oxidized regenerated cellulose
BCS: Breast conserving surgery
STIR: Short tau inversion recovery
QUORC: QUadrantectomy with oxidized regenerated cellulose
MRI: Magnetic resonance imaging
